# Liposomes to Augment Dialysis in Preclinical Models: A Structured Review

**DOI:** 10.3390/pharmaceutics13030395

**Published:** 2021-03-16

**Authors:** Kevin Hart, Martyn Harvey, Mingtan Tang, Zimei Wu, Grant Cave

**Affiliations:** 1 Intensive Care Unit, Hawkes Bay District Health Board, Hastings 9014, New Zealand; Grant.Cave@hbdhb.govt.nz; 2 Waikato Hospital Emergency Department, Waikato District Health Board, Hamilton 3240, New Zealand; martyn.harvey@waikatodhb.health.nz; 3 Faculty of Medicine and Health Sciences, School of Pharmacy, University of Auckland, Auckland 1023, New Zealand; m.tang@auckland.ac.nz (M.T.); z.wu@auckland.ac.nz (Z.W.)

**Keywords:** liposome, dialysis, ammonia, intoxication

## Abstract

In recent years, a number of groups have been investigating the use of “empty” liposomes with no drug loaded as scavengers both for exogenous intoxicants and endogenous toxic molecules. Preclinical trials have demonstrated that repurposing liposomes to sequester such compounds may prove clinically useful. The use of such “empty” liposomes in the dialysate during dialysis avoids recognition by complement surveillance, allowing high doses of liposomes to be used. The “reach” of dialysis may also be increased to molecules that are not traditionally dialysable. We aim to review the current literature in this area with the aims of increasing awareness and informing further research. A structured literature search identified thirteen papers which met the inclusion criteria. Augmenting the extraction of ammonia in hepatic failure with pH-gradient liposomes with acidic centres in peritoneal dialysis is the most studied area, with work progressing toward phase one trials. Liposomes used to augment the removal of exogenous intoxicants and protein-bound uraemic and hepatic toxins that accumulate in these organ failures and liposome-supported enzymatic dialysis have also been studied. It is conceivable that liposomes will be repurposed from the role of pharmaceutical vectors to gain further indications as clinically useful nanomedical antidotes/treatments within the next decade.

## 1. Introduction

Liposomes are spherical particles composed of a phospholipid bilayer encircling an aqueous centre [1]. First described in 1965, liposomes, particularly nano-sized liposomes, have since been widely investigated as drug-delivery carriers, and were the first nanomedicine platform to bridge the gap between in vitro studies and clinical use [2]. Liposomes have since become established as drug-delivery carriers [2]. In recent years a number of groups have been investigating the use of “empty” liposomes with no drug loaded as scavengers both for exogenous intoxicants (intoxicants) and endogenous toxic molecules (endogenous toxins), which accumulate due to diminished excretion in cases of organ failure. Preclinical trials have demonstrated that repurposing liposomes to sequester such compounds may prove clinically useful [3].

Studies have shown that intravascular “empty” liposomes can sequester compounds in vivo, thus acting as detoxification vehicles or “sinks” [4]. The first work in this field investigated detoxification in drug overdose and has since expanded into use in hepatic and renal failure. There are dose limitations to the intravenous (IV) dosing route, as many nanoparticles, including liposomes, can cause complement activation-related pseudoallergy (CARPA) when delivered intravenously [5,6]. This has led to the investigation of the use of liposomes in dialysate.

The predominant clinical use of dialysis is to facilitate the clearance of water-soluble toxic substances which accumulate in the blood of patients with renal failure. In clinical dialysis, blood flows over a semi-permeable membrane, with molecules moving into dialysate on the other side of the membrane via diffusion or convection [7]. Blood is never in contact with the dialysate. The pore size and configuration in the membrane of both peritoneal and haemodialysis (HD) does not allow the passage of the large complement proteins. The diameter of the main complement regulatory protein C3 is >100 Angstroms, compared to the pore size in dialysis of 5–50 Angstroms, which largely shields the components of dialysate from complement surveillance [8,9,10]. The addition of liposomes to dialysate in peritoneal dialysis (PD) or haemodialysis (HD) thus reduces the potential for CARPA, allowing for the protected introduction and removal of liposomes, which sequester the toxin. The use of liposomes in dialysate may also increase the reach of dialysis to molecules that were not previously removable.

PD uses the membrane lining the abdominal cavity, the peritoneum, as the dialysis membrane. Fluid introduced into the abdominal cavity dialyses against blood across the peritoneal membrane. PD is less invasive than HD, in which blood flows through an extracorporeal circuit across a dialysis membrane with countercurrent dialysate flow. In renal failure, HD is considered more effective than PD due to higher blood flows across the dialysis membrane, a higher ratio of dialysate to blood flow and the countercurrent dialysate flow in HD. PD is only used in approximately 10% of dialysed patients worldwide [11]. Increasing the effective “volume” of dialysate for any given molecule by entrapping the target molecule in liposomes suspended within peritoneal dialysate could increase the efficacy of PD to extract the target molecule. The basic principle involves introducing liposomes as a constituent of the dialysate in the patient’s peritoneal cavity, using the binding characteristics of the liposome to entrap the toxin of interest as it diffuses from blood perfusing the peritoneal cavity, then extracting the toxin-laden dialysate [1,4]. The use of liposomes in this way has been described as liposome-supported peritoneal dialysis (LSPD).

HD is currently used in some cases of poisoning, but limitations mean that dialysis is useful only in specific intoxications [12]. It is presently considered that only “free” toxins i.e., those that are not bound to plasma proteins, can by effectively dialysed. For that reason, in intoxications with drugs exhibiting protein binding of 80% or more, dialysis is not the usual treatment. Furthermore, compounds with a high volume of distribution also pose difficulties, as dialysis can only remove toxins contained in the blood compartment. Liposomes hold some promise as a means of addressing the first of these limitations and have been investigated as a means of ameliorating the second.

The use of lipid-based nanoparticles to augment dialysis is a nascent field with significant potential to improve clinical outcomes in a number of areas. In this review, we aim to provide a summary of current research to increase awareness and inform further research. As such, we present a review of the published literature on the use of liposomes to augment dialysis in preclinical models for both endogenous toxins and intoxicants. We focus most on repurposing the use of liposomes into new indications in areas of unmet clinical need.

## 2. Methods

We performed a literature search with the aim of identifying all published papers relating to the use of liposomes and lipid-based nanoparticles to augment dialysis in preclinical models for both endogenous toxins and intoxicants. We specifically included all experimental models both in vivo and in vitro, provided that the hypothesis tested in the study involved the capacity of liposomes to increase the extraction of target molecules into dialysate. Review papers were excluded.

The search involved both free text and Medical Subject Headings (MeSH) terms and included “((lipid and nanoparticle) or liposome) and (haemodialysis or hemodialysis or peritoneal dialysis)”. PubMed was searched from 1966 to November 2020. Google scholar was searched using the same search terms. Publications from any language were considered. There were 143 results returned on PubMed and 109 results on Google Scholar. We additionally searched the bibliographies of relevant papers, along with a PubMed search of prominent authors identified. Abstracts were reviewed by one author (KH), with subsequent review of full texts as necessary. Where any issues were identified around study inclusion, these were resolved by discussion between two authors (KH and GC). Studies were included if liposomes were used in vitro or in vivo to augment dialysis of either endogenous toxins or intoxicants. Given the small number of papers and the heterogeneity of targets, liposomes and experimental models used, we extracted a summary of the experimental design with outcomes relevant to that design from the identified papers.

## 3. Results

**Figure 1:**
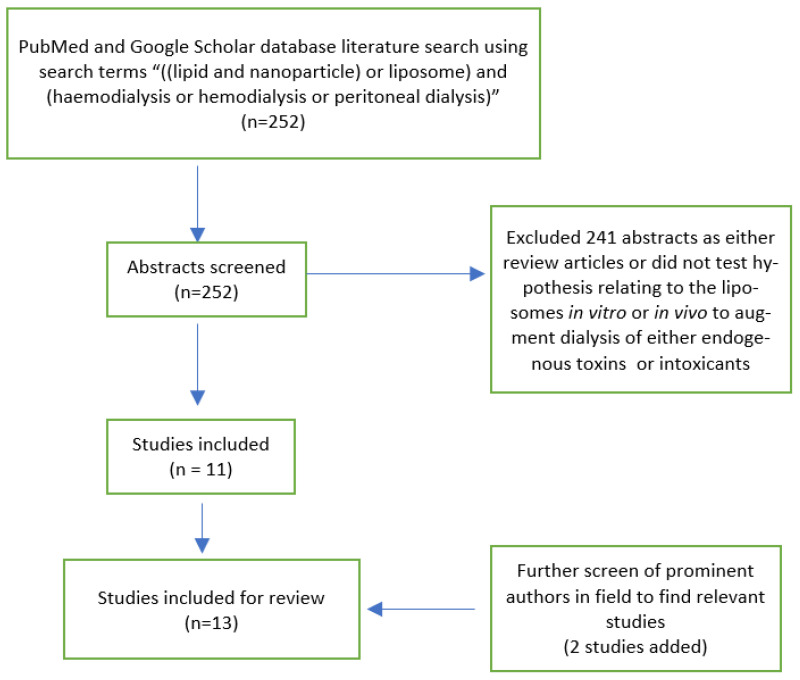
PRISMA diagram of studies included in this review.

The data search identified 252 citations, although some of these were dual citations identified in both search engines. Of the 252 citations, 11 studies were identified where a hypothesis was tested involving the use of liposomes in vitro or in vivo to augment dialysis of either endogenous toxins or intoxicants. An additional two studies were identified (Figure 1).

As no studies involving lipid-based particles other than liposomes were found, we refer to liposomes only in the review. The use of liposomes to increase the extraction of endogenous toxins and intoxicants has been investigated in 13 studies. The use of pH-gradient liposomes with an acidic core to increase the extraction of ammonia is the most investigated area. Improving dialysis of the protein-bound endogenous toxins relating to renal failure with soy phospholipid (SP)-based liposomes, cationic liposomes and liposomes made with linoleic acid was the subject of four studies. Three studies related to intoxicants, investigating the capacity of liposomes to augment dialysis for verapamil, amitriptyline, haloperidol, propranolol and phenobarbitone. One study evaluated liposome-supported enzymatic peritoneal dialysis, and one study evaluated the effects of SP liposomes on dialysis of protein-bound endogenous toxins, which accumulate in hepatic failure.

A summary of studies with experimental models, liposome type and targets, including exogenous and endogenous toxic substances, and results relevant to the model is shown in Table 1.

## 4. Discussion

The use of liposomes in dialysate is in varying stages of preclinical development and is focused on areas of currently unmet clinical need. Liposomes in dialysate may increase the “reach” of dialysis for protein-bound molecules in blood and increase the total extraction capacity of peritoneal dialysate for ammonia. Larger doses of liposome are able to be used in dialysate than could be given intravenously as dialysate is in a compartment separated from complement surveillance. It is conceivable that liposomes will be repurposed from the role of pharmaceutical vectors to gain further indications as clinically useful nanomedical antidotes/treatments within the next decade.

Discussions categorised by the class of liposome target are outlined below.

### 4.1. Endogenous Toxic Substances

Ammonia is an endogenous metabolite produced primarily from the breakdown of amino acids. It is taken up by the liver and converted in the urea cycle, but accumulates in inborn errors of metabolism and hepatic failure. The accumulation of ammonia in hepatic failure is pathogenic in the cerebral oedema seen with this condition. Ammonia is a lipid-soluble weak base and, as such, represents a rational target for entrapment by pH-gradient liposomes with an acidic centre. Forster et al. used LSPD to entrap ammonia in rat models with greater efficacy than conventional PD [11]. Agostoni et al. used pH-gradient liposomes in LSPD to treat hyperammonemia in rat models of hepatic cirrhosis [14]. This technique resulted in a 10-fold increase in dialysate ammonia removal compared with conventional PD and reduced both ammonia concentrations in the plasma and the degree of brain oedema. Liposomes in this experiment were prepared using an osmotic shock technique, whereby liposomes suspended in water were “shocked” by the sudden addition of citric acid to achieve pH 2. The sudden osmotic change induced transient increased permeability in the liposome membrane with subsequent pH equilibration. The suspensate was then neutralised with an alkaline xylitol-based solution. Preparation of liposomes in this way could markedly increase the storage life, with pH neutralisation as the last step prior to use. Giacalone et al. demonstrated in 2018 that pH-gradient liposomes maintained their ability to sequester ammonia in vitro in ascitic fluid from patients with liver disease when co-incubated with drugs commonly administered to this patient group such as beta-blockers and diuretics [15]. In the same study it was demonstrated that LSPD did not remove more essential endogenous metabolites than conventional PD fluid.

Matoori et al. investigated the pharmacokinetics and safety profile of LSPD using pH-gradient liposomes with acidic centres in dialysate in healthy minipigs [16]. This study was undertaken with a view toward meeting safety requirements for approval for a first-in-human study. Two doses of liposomes were administered intraperitoneally daily for ten days to healthy minipigs, as well as in a pig model of hyperammonaemia. The results demonstrated the pharmacokinetics of citric acid (the driver of the liposome core pH gradient) to be linear and the minipigs showed only low systemic accumulation of phospholipids. In addition, there were no complement-activation-related pseudoallergy reactions and the LSPD was tolerated well. Finally, in a hyperammonaemic pig model, pH-gradient liposome-containing dialysate was found to have significantly higher levels of ammonia in the peritoneal fluid when compared with the liposome-free control group [16]. Given that hyperammonemia carries a poor prognosis in liver failure, with the risk of encephalopathy, coma and death, and that there are limited treatment strategies available, LSPD represents a promising treatment avenue. The website of the patent-holders for this technology lists their pH-gradient with acidic centre liposomes in dialysate as being at the phase 1 stage of preclinical development.

### 4.2. Endogenous Protein-Bound Solute Dialysis

Protein-bound uremic toxins (PBUTs) such as p-cresyl sulphate (PCS) and indoxyl sulphate (IS), which are products of bacterial intestinal amino acid metabolism, accumulate in end-stage renal disease. Their accumulation is important because, although PBUTs are not the cause of initial renal failure, PBUT concentrations are associated with increased mortality and morbidity in long-term haemodialysis patients. Increased PBUT concentrations are also associated with higher rates of vascular complications and more rapid progression of renal failure. Their clearance by traditional HD and PD is poor due to their high protein binding in plasma [17]. In order for PBUT clearance by dialysis to be increased by a nanoparticle, PBUTs must first dissociate from their binding protein into the free fraction in plasma, diffuse from the bloodstream into the dialysate and subsequently be bound in the dialysate to maintain a concentration gradient for the toxin to continue leaving the bloodstream. This process must also occur within the span of a capillary (for PD) or dialysis cartridge (for HD) transit time [18]. Preliminary studies imply that adding albumin or soy phospholipid-based liposomes as binders to dialysate could improve PBUT removal compared with conventional glucose-based peritoneal dialysis [18]. The addition of soy phospholipid-based liposomes to the dialysate in haemodialysis for uraemic rats also increased PBUT clearance [17].

Strategies to improve the extraction capacity of liposomes in dialysate for PBUTs have been evaluated. Liposomes loaded with molecules that compete with PBUTs for albumin binding sites, thus displacing PBUTS and increasing the free fraction concentration in the blood available for dialysis, have been investigated. Linoleic acid-modified liposomes (LA-liposomes) were used in the dialysate and assessed for their capacity to increase PBUT clearance [19]. As previously discussed, toxins not in the free fraction are inaccessible to traditional dialysis. It was shown that using linoleic acid as a “displacer” to compete with PBUTs for albumin binding sites significantly increased the clearance of PBUTs in in vitro dialysis models. The same effect was observed for LA liposomes for liver failure-related cholestatic solutes.

PCS and IS are molecules based on an aromatic ring with a negatively charged sulphate group attached. Liposomes bind PBUTs via electrostatic and lipophilic interactions with the liposome membrane. A positively charged amphiphile on the liposome phospholipid was trialed to increase liposome affinity for PBUTs, with increased extraction of IS and p-Cresol using an in vitro dialysis model [20].

Preclinical work has identified investigational targets other than ammonia for liposomes to augment dialysis in the management of liver failure. Both unconjugated bilirubin and bile salts were found to have significantly higher reduction ratios with liposome dialysis when compared to conventional dialysate using in vivo haemodialysis models [21].

### 4.3. Exogenous Toxic Substances

pH-gradient liposomes with acidic cores sequester lipophilic weak bases via the mechanism of ion trapping [22]. Peritoneal dialysis-medium-containing acidic core pH-gradient liposomes have been demonstrated to extract ionisable drugs and toxins in animal models. As early as 2012, a group investigated using drug-scavenging acidic-centre pH-gradient liposomes to treat calcium channel blocker (CCB) overdose [5]. Given the vascular and cardiac effects of drugs such as verapamil, these drugs are potentially life-threatening when ingested in an overdose. Current conventional decontamination methods are not reliably effective and efforts to establish a definitive antidote remain unsuccessful. Forster et al. initially showed that pH-gradient liposomes could sequester CCBs in vitro, before successfully demonstrating that IV injection of these liposomes into rats intoxicated with verapamil reversed the cardiovascular effects of intoxication. Although this study was an early step in showing the viability of liposome use in intoxication, issues of CARPA and low sequestration capacity relative to total toxic dose ingested remained. These limitations are mitigatable through introducing liposomes into dialysate, where liposomes are introduced to a compartment free from complement surveillance and intoxicant entrapped in liposomes can be removed from the body.

This same group developed LSPD using acidic-centre pH-gradient liposomes [7] in dialysis medium to increase dialytic clearance of target molecules using the lining of the abdominal cavity, the peritoneum, as the dialysis membrane. This group demonstrated the ability of liposomes in LSPD to sequester ionisable drugs via peritoneal dialysis in a rat model. Dialysate could then be extracted from the peritoneal cavity to eliminate the drug. The extraction of multiple drugs, including verapamil, propranolol, haloperidol and amitriptyline, was demonstrated. Of note, most of these drugs do not have specific antidotes and all are common presentations in overdose. LSPD removed 80 times more verapamil than in conventional dialysate controls. As in the previous study using IV administered liposomes, this method successfully demonstrated a reversal of the cardiovascular effects of verapamil in a rat model of verapamil overdose. The mechanism of entrapment was the same as for ammonia, with the lipophilic, weakly basic verapamil diffusing through the liposome membrane, then becoming protonated in the liposome centre and thus unable to exit through the liposome wall. This created a much larger “sump” for verapamil than regular dialysate, and therefore performed continuous extraction of verapamil from blood perfusing the peritoneal cavity. Of note, one of the further findings of this study was that smaller liposomes, 200 nm in diameter, had markedly greater systemic absorption from the peritoneal cavity than liposomes 800 nm in diameter.

Chapman et al. investigated whether the presence of acidic-centre liposomes in peritoneal dialysate could increase amitriptyline extraction via trans-liposomal pH gradient into dialysate in rats [23]. Based on estimates of peritoneal blood flow, the authors were the first to postulate that liposomes used in this way were effective in increasing the dialysis of protein-bound solutes in vivo. The same group have also investigated whether increasing blood concentrations of amitriptyline by adding either intravenous lipid emulsion (ILE) [23] or IV liposomes with the anionic phospholipid 1,2-dioleoyl-*sn*-glycero-3-phosphoglycerol (DOPG), which bind amitriptyline in the membrane, and would increase LSPD drug elimination [24]. These approaches represented an attempt at using liposomes or ILE to mitigate both of the major limitations to dialysis in intoxication—IV liposomes/emulsion to mitigate the problem of low relative blood concentrations, with intraperitoneal liposomes to increase dialysis of a bound solute. Although LSPD seems effective in increasing the removal of protein-bound solute into dialysate, no effect from increasing blood concentrations using ILE or DOPG liposomes has been observed.

### 4.4. Enzymatic Liposome Dialysis

Liposome-supported enzymatic peritoneal dialysis (LSEPD) is the use of enzyme-loaded liposomes (E-liposomes) to increase the functionality of PD. This technique was investigated in a rodent ethanol intoxication model [25]. Liposomes loaded with alcohol oxidase and catalase increased ethanol metabolism when compared with the free enzymes in dialysate, as evidenced by any increase in ethanol’s primary metabolite, acetaldehyde, in both plasma and dialysate. E-liposomes also displayed less systemic exposure and organ distribution than the free enzymes in dialysate. Although LSEPD did not show significantly lower systemic ethanol levels in these rodent models [26], this work demonstrates that the peritoneal cavity could be utilised as an epicentre for these chemical reactions. Future work may focus on using this technique to increase the systemic clearance of target molecules, such as toxic alcohols.

### 4.5. Liposome Formulation and Modifications

In addition to classification by the origin of the target molecule, work in this area can be viewed based on the liposome formulation and molecular structure of the target molecule. Forster et al., in their initial work using IV liposomes, undertook initial in vitro work to evaluate which formulation had the greatest binding capacity to bind diltiazem in suspensions of fetal bovine serum. Both intravenous lipid emulsion and anionic dipalmitoylphosphatidylcholine (DPPC) liposomes entrapped diltiazem, a lipophilic weak base. Diltiazem uptake by DPPC liposomes increased by a factor of 14 as the pH of the aqueous centre dropped from 7.4 to 2, which is consistent with the principle of ion trapping [5]. This in vitro finding, subsequently replicated for other lipophilic weak bases [11], has translated into positive in vivo experimental results using pH-gradient liposomes for ammonia, verapamil and other molecules.

Target molecules with different molecular structure have been sequestered in dialysate via electrostatic and lipophilic interactions in the liposome membrane. The in vitro refinements of liposome structure to sequester PBUTs using cationic phospholipid and linoleic acid to displace PBUTs from albumin are yet to translate to in vivo findings.

### 4.6. Challenges/Perspective

The potential for the addition of liposomes into dialysate to improve clinical management has previously been noted [1]. Although this field holds promise for improvement in treating conditions where there is unmet need, challenges remain. LSPD for hepatic failure is furthest along the development pipeline, though further development hurdles remain. The shelf life of liposomes for use in dialysate is an issue which may impair uptake, though the osmotic shock technique for the preparation of acidic centred liposomes may mitigate this. In dialysis to treat renal failure, the concentration of liposomes used experimentally to increased PBUT extraction is high and may not be transferrable to the clinical situation. Liposome-supported enzymatic dialysis is a very nascent field, requiring further research.

A counterbalance to these limitations on development is the scope of unmet clinical need which liposomes in dialysate may address. With 3 million patients worldwide receiving haemodialysis [27], receiving at least 2 treatments per week, there are in the order of 300 million yearly treatment episodes with potentially improved PBUT extraction using liposomes in dialysate. The developers of LSPD to treat hyperammonaemia in liver failure similarly estimate a significant potential unmet need to drive development [28].

## 5. Conclusions

Although the use of liposome-supported peritoneal dialysis remains in the preclinical phase, there are promising signs for its potential use in areas of unmet clinical need in the future. The use of LSPD to increase ammonia extraction is the indication that is furthest along the development pipeline. The use of liposomes to improve the clearance of protein-bound uraemic toxins in renal failure is a nascent investigational field, which could potentially augment treatments for a large population with a chronic illness. Experiments in which liposomes are used in dialysate appear to mitigate the problems encountered using intravenous liposomes and traditional dialysis techniques in animal models for specific intoxications. It is conceivable that liposomes will be repurposed from the role of pharmaceutical vectors to gain further indications as clinically useful nanomedical antidotes/treatments within the next decade.

## Figures and Tables

**Table 1:** Summary of studies included in the review.

Authors	Experimental Model	Target Molecule	Liposome Type	Results: In Vitro	Results: In Vivo
Exogenous toxic substances					
Forster et al. (2014)	pH-gradient liposomes in vitro and LSPD in vivo (rat model) Pharmacodynamic endpoints also for verapamil in vivo (rat model)	Verapamil, Propranolol, Amitriptyline, Phenobarbital	Liposome with acidic core (except phenobarbital where core basic) Liposomes bind via ionisation of weak base/acid in liposome core to become lipid insoluble, thus entrapped	90% of verapamil was sequestered in Liposomes within 8 h in presence of plasma proteins	Verapamil: >80-fold increased extraction of drug over a 12-h peritoneal dwell time versus conventional icodextrin dialysate control. ^†^ LSPD in a rat model of verapamil intoxication resulted in 3-fold reduction in time to blood pressure recovery. ^†^ Other drugs: LSPD in vivo showed increased extraction versus conventional dialysate: Phenobarbital 1.25-fold, haloperidol > 100-fold, propranolol > 100-fold. ^†^
Chapman et al. (2019)	LSPD in vivo ± ILE (rat models)	Amitriptyline	Liposomes with acidic core	Nil	12-fold increase in dialysate amitriptyline concentration with LSPD. ^†^ Hypothesis generating finding of estimated extraction ratio for amitriptyline from peritoneal blood flow for LSPD 30%—much greater than reported free fraction amitriptyline (5–10%) [13]
Cave et al. (2018)	LSPD in vivo ± IV DOPG liposomes (rat models)	Amitriptyline	Liposomes with acidic core for liposomes in peritoneal dialysate IV DOPG liposomes (DOPG: 1,2-dioleoyl-*sn*-glycero-3-phosphoglycerol) DOPG liposomes bind via electrostatic/lipophilic interaction in liposome membrane	Nil	DOPG liposomes increased blood amitriptyline concentrations by 50%. No corresponding increase in LSPD dialysate amitriptyline concentrations at the end of the dwell time; control median 430 nmol/L vs. IV DOPG 414 nmol/L.
Enzymatic liposome dialysis					
Pratsinis et al. (2017)	LSPD in vivo (rat models)	Ethanol	Enzyme-loaded Liposomes (containing ethanol metabolising enzymes) compared with free enzymes in dialysate	Nil	E-liposomes enhanced ethanol metabolism, as evidenced by increased ethanol metabolites in both plasma and dialysate.
Endogenous toxic substances					
Forster et al. (2014)	LSPD both in vitro and in vivo (rat model)	Ammonia	Liposomes with acidic core	Liposomes extracted 95% of ammonia added to an in vitro diffusion system in 8 hr. When serum was added to mimic physiological conditions, the uptake into liposomes exceeded the total ammonia added.	20-fold increase in ammonia concentration in dialysate with a 3-hr. LSPD treatment in rats. ^†^
Agostoni et al. (2016)	In vitro: study on effect of concentration of citrate in liposome on ammonia removal capacity. In vivo: LSPD in rat and pig models	Ammonia	Liposomes with acidic core Large liposomes, prepared by osmotic shock technique	Acidic core of liposome confirmed as an influx driver of ammonia to liposomes, proportional to concentration of citrate in liposome.	One week of LSPD in rat models of induced hepatic failure: 10-fold increase in dialysate ammonia, reduced plasma ammonia concentration and brain water concentration versus conventional peritoneal dialysis. ^†^ LSPD with liposomes from this experiment did not cause CARPA in pigs.
Giacalone et al. (2018)	In vitro: capacity of liposomes for ammonia uptake and drug interactions in human ascitic fluid was assessed; in vivo: LSPD to assess uptake of important metabolites in a rat model	Ammonia	Liposomes with acidic core	LSPD maintained its ammonia uptake when combined with ascitic fluid from liver disease patients, with limited interaction effects when combined with drugs commonly co-administered to this patient group, except the lipophilic weakly basic propranolol and fluoroquinolones	LSPD did not remove important metabolites more than conventional PD fluid
Matoori et al. (2020)	LSPD in vivo (minipig models) Experiment undertaken to ascertain safety of the model prior to first in human study	Ammonia	Liposomes with acidic core	Nil	Increased dialytic clearance of ammonia in ammonium chloride infusion with LSPD. ^†^ LSPD model in pigs demonstrated low plasma citrate (driver of liposome pH gradient) concentrations and, low DPPC absorption and no CARPA with daily doses for 10 days (safety endpoints).
Endogenous bound solutes					
Shi et al. (2019)	Liposomes both in vitro and an in vivo rat peritoneal dialysis model Albumin both in vitro and an in vivo rat periotneal dialysis model	p-cresyl sulphate (PCS), indoxyl sulphate (IS) and indole-3-acetic acid (3-IAA)	Soy phospholipid liposomes Mechanism of binding electrostatic/lipophilic interaction in liposome membrane	Adding liposomes or albumin to dialysate markedly increased removal of PCS and IS. Albumin Markedly increased removal of 3-IAA.	Both LSPD and albumin resulted in higher concentrations of intraperitoneal PBUTs. ^†^
Shi et al. (2019)	Liposome-supported HD both in vitro and in vivo (rat models)	PCS, IS and Hippuric acid (HA)	Soy phospholipid liposomes	Percentage removal of both PCS and IS, but not HA, increased as the liposome dose increased in a dose-response relationship. Adding liposomes to dialysate markedly increased removal of PBUTs without significantly altering urea and creatinine clearance	Adding liposomes resulted in higher reduction ratios and more total solute removal for several PBUTs when compared to conventional dialysate. ^†^
Shen et al. (2020)	In vitro—rapid equilibrium dialysis (RED) device.	PCS and IS	Cationic liposomes (modified) vs. SP liposomes Cationic liposome exhibit increased electrostatic attraction in membrane	Cationic showed higher binding rate with IS (1.24–1.38 fold higher) and PCS (1.07–1.09 fold higher) compared with plain liposomes. Cationic liposome-supported dialysis had a better clearing efficiency for PCS and IS when compared with conventional dialysate and with dialysate containing bovine serum albumin.	Nil
Shen et al. (2020)	Liposome-supported haemodialysis both in vitro and in vivo (rat model)	Unconjugated bilirubin and bile salts	Soy phospholipid liposomes	Unconjugated bilirubin (52.83%–99.87%) and bile salts (50.54%–94.75%) were bound by liposomes in a dose–response relationship. Concentrations of both were significantly decreased in the liposome dialysis group vs. phosphate buffered saline group.	Liposome-containing dialysate resulted in a significantly higher reduction ratio in total bilirubin (6.56% ± 5.72% vs. −1.86% ± 5.99%, *p* < 0.05) and more total bile acids (7.63 ± 5.27 μmol vs. 2.13 ± 2.32 μmol, *p* < 0.05) extracted compared with conventional dialysate. ^†^
Shen et al. (2020)	In vitro dialysis (Ultrafiltration column)	representative PBUTs and liver failure-related solutes	Linoleic acid-modified liposomes (LA-liposomes) Linoleic acid acts as displacer of target molecule from albumin to increase free fraction available	LA-liposomes exhibited binding to PBUTs, bilirubin and bile acids. Dialysate containing LA-liposomes showed higher reduction rates of these compounds compared with traditional dialysate and when compared with dialysate containing plain liposomes. Additionally, adding LA resulted in the significant inhibition of albumin binding PBUTs, so removal efficiency of PBUTs was greatly enhanced with LA as a competitive displacer.	Nil

Table abbreviations: CARPA—complement activation-related pseudoallergy; DOPG—1,2-dioleoyl-*sn*-glycero-3-phosphoglycerol; DPPC—dipalmitoylphosphatidylcholine; HA—hippuric acid; ILE—intravenous lipid emulsion; IS—indoxyl sulphate; IV intravenous; LA—linoleic acid; LSPD—liposome-supported peritoneal dialysis; PBUT—protein-bound uraemic toxin; PCS—p-cresyl sulphate; SP—soy phospholipid. ^†^ indicates a positive in vitro finding which translated into a positive in vivo finding.
